# Human Monocyte Heat Shock Protein 72 Responses to Acute Hypoxic Exercise after 3 Days of Exercise Heat Acclimation

**DOI:** 10.1155/2015/849809

**Published:** 2015-03-22

**Authors:** Ben J. Lee, Richard W. A. Mackenzie, Valerie Cox, Rob S. James, Charles D. Thake

**Affiliations:** ^1^Exercise Science Applied Research Group, Coventry University, Priory Street, Coventry CV1 5FB, UK; ^2^Inflammation and Infection Group, School of Science and Technology, University of Westminster, New Cavendish Street, London W1W 6UW, UK

## Abstract

The aim of this study was to determine whether short-term heat acclimation (STHA) could confer increased cellular tolerance to acute hypoxic exercise in humans as determined via monocyte HSP72 (mHSP72) expression. Sixteen males were separated into two matched groups. The STHA group completed 3 days of exercise heat acclimation; 60 minutes cycling at 50% $\dot{\text{V}}{\text{O}}_{2\text{peak}}$ in 40°C 20% relative humidity (RH). The control group (CON) completed 3 days of exercise training in 20°C, 40% RH. Each group completed a hypoxic stress test (HST) one week before and 48 hours following the final day of CON or STHA. Percentage changes in HSP72 concentrations were similar between STHA and CON following HST1 (*P* = 0.97). STHA induced an increase in basal HSP72 (*P* = 0.03) with no change observed in CON (*P* = 0.218). Basal mHSP72 remained elevated before HST2 for the STHA group (*P* < 0.05) and was unchanged from HST1 in CON (*P* > 0.05). Percent change in mHSP72 was lower after HST2 in STHA compared to CON (*P* = 0.02). The mHSP72 response to hypoxic exercise was attenuated following 3 days of heat acclimation. This is indicative of improved tolerance and ability to cope with the hypoxic insult, potentially mediated in part by increased basal reserves of HSP72.

## 1. Introduction

Heat acclimation induces an increase in basal stores of the evolutionarily conserved molecular chaperone heat shock protein 72 (HSP72) [1, 2]. Additionally, HSP72 is induced by exposure to hypoxia at rest in humans [3]. These data demonstrate a degree of commonality in stress adaption and thus the potential to exploit cross acclimation in humans in preparation for exposures to different physiological stressors.

An increase in the basal stores of HSP72 represents an improvement in a cell's ability to tolerate stress without the need for* de novo* protein synthesis [4] and is an accepted marker in an organism's adaptation to stress [5]. It is possible that invoking the heat shock response (HSR) via exposure to one stress may induce a degree of tolerance to a second, different stressor [6]. This cross acclimation is well documented* in vivo* and* in vitro* in animal models (for a review see Horowitz, 2007 [6]). For example, hemodynamic recovery is enhanced in heat acclimated animals exposed to a hypoxic stressor (such as ischemia reperfusion) compared to control animals [7]. However, to date very little is known about the evocation of acclimation* in vivo* in humans. Taylor et al. [8] indicated that increased basal stores of monocyte HSP72 (mHSP72) during 5 daily resting hypoxic exposures were associated with reduced oxidative stress after submaximal exercise in normoxia. However the absence of normoxic [8] or normothermic [1] controls in human* in vivo* studies often makes it difficult to determine whether the intervention alone led to the increase in mHSP72.

HSP72 can be released into the circulation in response to stress and may serve as part of the immune response [9, 10]. Changes in circulating HSP72 (eHSP72) have been proposed to have effects on the cytokine cascade and therefore affect the inflammatory response* in vivo* [11]. eHSP72 is released following exercise in an intensity and duration dependent manner [12, 13] and heat acclimation has been shown to reduce basal eHSP72 and attenuate eHSP72 responses after subsequent exercise-heat stress [14–16]. However both the tissue of release and physiological relevance of eHSP72 release during exercise and following heat acclimation remain unclear. Furthermore, the response of eHSP72 to hypoxic exercise in humans remains undefined in the literature.

Heat acclimation is a complex process involving actions at both the whole body and the cellular level [17]. Regimens for humans are traditionally medium (8–14 days) or long term (>15 days) in their nature [18]. It is now accepted that many of the beneficial adaptations to heat stress are cardiovascular in nature, for example, reduced exercising heart rate (HR) and core temperature (*T*_core_) and increased sweat rates, and occur rapidly over the initial 3–5 days of acclimation [19]. A 3-day heat acclimation protocol has also been shown to increase the basal levels of HSP72 mRNA alongside small increases in HSP72 protein [14]. Therefore shorter term protocols may be more appropriate and logistically easier to implement in preparation for exposure to hypoxic-based stressors.

To date, no research has examined whether in humans the initial phase of heat acclimation and the associated increase in basal levels of HSP72 may confer tolerance to a subsequent exposure to a different stressor. Thus the primary aim of this study was to determine whether short-term heat acclimation (STHA) in humans could induce an increase in basal mHSP72 and precondition against a subsequent bout of acute hypoxic exercise when compared to a normothermic control group. Secondly, the eHSP72 response to acute hypoxic stressors before and after STHA was examined. It was hypothesized that 3 days of heat acclimation would increase basal stores of mHSP72 and that the increased availability of mHSP72 would attenuate the response of this cytoprotective protein following a bout of acute hypoxic exercise.

## 2. Methods

### 2.1. Participant Characteristics

**Figure 1:**
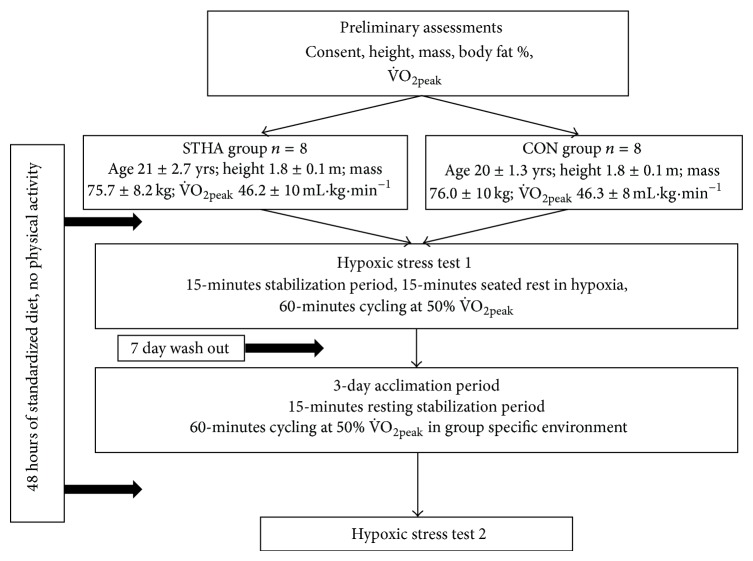
Experimental schematic. See text for details. STHA = short-term heat acclimation; CON = control.

Sixteen healthy males provided signed informed consent prior to participation in this study, which was granted approval by the Coventry University Ethics committee, and were divided into 2 matched groups based on their aerobic capacity (Figure 1). All were physically active, nonsmokers with no prior history of cardiorespiratory illness. Laboratory attendance time was kept constant within each participant in order to minimize the effects of circadian variation on performance and the known diurnal variation in mHSP72 [20]. Caffeine [21] and alcohol consumption were barred from all meals and beverages for 72 h prior to each laboratory visit. Participants were required to maintain a food and activity diary as accurately as possible for 3 days prior to each experimental visit and then requested to replicate this prior to subsequent visits [22]. Additionally, participants refrained from all supplementation (i.e., vitamins, ergogenic aids) throughout the study period. Participants were requested to abstain from prolonged thermal exposures (baths, saunas, steam rooms, and tanning devices) and vigorous physical activity for seven days prior to the preliminary testing and throughout the remaining experimental program. Participants who had visited or resided at altitudes in excess of 1000 m [3] or climates with ambient temperatures in excess of 30°C [23, 24] or had experienced high pressure environments, for example, hyperbaria, within the three months prior to study commencement were excluded during recruitment due to the possible influence of such environments on basal HSP72 expression. Participants fasted for 2 hours prior to each trial and did not eat until the final blood withdrawal of each trial. Compliance for all the aforementioned experimental controls was monitored via questionnaires administered before, during, and after the extended experimental study period and was reported as 100% in all participants.

### 2.2. Experimental Design

Participants reported to the laboratory on six occasions, outlined in Figure 1. The first involved assessment of preliminary measures of anthropometry, lactate threshold, and $\dot{\text{V}}{\text{O}}_{2\text{peak}}$. Participants then returned 7 days later to undergo an exercise hypoxic stress test (HST; visit 2). At least 7 days after the HST participants completed 3 days of either STHA or control acclimation (CON; visits 3, 4, and 5) and returned 48 hours after the final acclimation session to complete a final HST (HST2, visit 6). A fractional inspired oxygen level of 0.14 (equivalent to ~3000 meters above sea level) was selected for all hypoxic trials and heat acclimation temperature of 40°C for the STHA group as they are close to the acute habitable limits for nonacclimatized individuals. These environmental conditions were chosen to reflect conditions regularly experienced on sojourns by athletic populations, adventure tourists, and the military.

### 2.3. Visit 1: Preliminary Testing

The initial visit involved preliminary tests for resting hemoglobin concentration, anthropometry to measure height, weight, and estimated body fat followed by the measurement of lactate threshold and peak oxygen uptake ($\dot{\text{V}}{\text{O}}_{2\text{peak}}$).

Peak oxygen uptake was determined using an incremental exercise test to volitional exhaustion on a cycle ergometer (Monark Ergomedic 874e, Monark Exercise AB, Vansbro, Sweden) whilst breathing room air. Resting blood lactate (Biosen C-Line Analyser, EKF Diagnostics, Germany) was determined from a finger capillary whole blood sample following a 10-minute seated rest period. The test began at a workload of 70 W for 4 minutes and was then increased by 35 W every 4 mins until a blood lactate value of >4 mmol·L^−1^ was reached. Thereafter, workload increased 35 W every 2 minutes until volitional exhaustion. A cadence of 70 rev·min^−1^ was maintained throughout. Expired gas was collected into 200 L Douglas bags between minutes 3 and 4 of each 4-minute stage and then minutes 1-2 of each 2-minute stage. Expired gas samples were analyzed to determine CO_2_ and O_2_ content, using a Servomex infrared and paramagnetic gas analyzer, respectively (model 1400, Servomex, Crowthorne, UK), and gas volume, via a Harvard Dry Gas meter (Cranlea and Company, Birmingham, UK). Peak oxygen consumption was considered to be achieved if at least two of the following criteria were met: (i) a respiratory exchange ratio of >1.1, (ii) a heart rate greater than 95% of age predicted maximum (220-age), and (iii) a final blood lactate value in excess of 8 mmol·L^−1^. This protocol has shown a CV of <1.5% for oxygen consumption in our laboratory.

### 2.4. Participant Preparation

The remaining 5 laboratory visits consisted of identical procedures and measurements. Upon arrival to the laboratory participants voided their bladder to provide a sample for assessment of urine specific gravity (USG; Visual Refractometer, Index Instruments, Cambridge, UK) and osmolality (Osmocheck, Vitech Scientific, Partridge Green, West Sussex, UK). Participants were considered euhydrated if these values were <1.030 and 600 mOsmol·kg^−1^, respectively [25]. They then measured their own nude body mass (Seca 899 scales, Seca, Hamberg, Germany), inserted a rectal thermometer 10 cm past the anal sphincter (Grant Instruments, Shepreth, UK), and attached a heart rate monitor to their chest (Suunto T6c, Suunto, Vaanta, Finland). Whilst seated, skin thermistors (Grant Instruments, Shepreth, UK) were fitted to the upper arm, upper thigh, chest, and calf using a micropore tape to enable continuous monitoring of skin temperature. Participants were then seated on a cycle ergometer (Monark Ergomedic 874e, Monark Exercise AB, Vansbro, Sweden) and completed a 15-minute resting period. At the end of the rest period a 7 mL venous sample was collected from an antecubital vein into potassium coated EDTA vacutainers (VACUETTE, Greiner Bio-One, Stonehouse, UK) for the immediate assessment of monocyte HSP72 (mHSP72). Heparinized capillary sample tubes were collected in triplicate and centrifuged (Hawksley Micro Hematocrit Centrifuge, Hawksley & Sons Ltd., UK) and hematocrit was determined by reading from the hematocrit reader (Hawksley Micro). Hemoglobin was determined via a calibrated B-Hemoglobin Photometer (Hemocue Ltd., UK) and corrected plasma volume was calculated [26].

### 2.5. Exercise Measurements

Participants completed 60 minutes of cycle exercise at an intensity corresponding to 50% normoxic $\dot{\text{V}}{\text{O}}_{2\text{peak}}$ in each of the 5 testing sessions. Measurement of heart rate, core and skin temperatures, arterial oxygen saturation (SpO_2_) and recording of RPE [27], and thermal sensation (TS; [28]) were noted at the end of rest after the venous blood withdrawal and at 10-minute intervals throughout the exercise period. Arterial Hb oxygen saturation (SpO_2_) was recorded during respiratory gas collections using a finger-clip pulse oximeter (3100 WristOx, Nonin Medical, Inc., Plymouth, MN, USA). The sensor has a reported accuracy of ±2 digits (manufacturers guide). Physiological strain was calculated using the physiological strain index (PSI; [28]). Sweat losses were determined by the change in nude body mass before and after exercise, with sweat volume and mass assumed as being the same (i.e., 1 mL = 1 g; [1]). Upon completion of each exercise bout a final venous sample (7 mL) was collected while participants remained on the cycle ergometer as previously described.

### 2.6. Visits 2 and 6: Hypoxic Tolerance Test

At least 5 days after the preliminary visit, participants returned to the laboratory for the baseline hypoxic stress test (HST1, visit 2 [29]). This procedure was repeated 48 hours after the final acclimation session (HST2, visit 6). After instrumentation, participants were seated on a cycle ergometer (Monark Ergomedic 874e) and completed a 15-minute normoxic resting period whilst breathing through a mouthpiece and 30 mm diameter connector (Harvard Ltd, Eldenbridge, UK) attached to a two-way nonrebreathable valve (Harvard Ltd, Eldenbridge, UK). Ethylene clear vinyl tubing was used to connect the inspiratory side of the valve to a series of 1000 L Douglas bags. During all hypoxic trials the 1000 L Douglas bags were filled with hypoxic gas (F_I_O_2,_ = 0.14) generated by an oxygen filtration device (Hypoxico HYP123 hypoxicator, New York, USA) prior to the start of all testing. After the initial 15-minute normoxic rest period participants completed a further 15-minute resting wash-in period in hypoxic conditions and 60 minutes of cycle exercise at an intensity corresponding to 50% normoxic $\dot{\text{V}}{\text{O}}_{2\text{peak}}$. Physiological and subjective variables were collected after each 15-minute rest period and every 10 minutes throughout exercise. Venous samples were collected as previously described at the end of the normoxic rest period and immediately upon completion of exercise. Exercise was terminated if arterial oxygen saturation fell below 70% and heart rate reached 95% $\dot{\text{V}}{\text{O}}_{2\text{peak}}$ for 3 consecutive minutes or if the participant requested to stop the trial.

### 2.7. Visits 3, 4, and 5: Intervention Period

At least 1 week after the initial HST, participants reported to the laboratory to undergo 3 days of heat acclimation (40°C, 20% RH; STHA) or exercise training (18°C, 20% RH; CON). Venous samples were collected as previously described on day 1 and day 3 after a 15-minute seated rest period outside the heat chamber. A final venous sample was collected with participants remaining seated on the cycle ergometer as soon as exercise was terminated. Participants entered the environmental chamber and completed a further 15-minute rest period before commencing cycling at 70 rpm and against a resistance sufficient to elicit 50% normoxic $\dot{\text{V}}{\text{O}}_{2\text{peak}}$ for 60 minutes [30, 31]. Physiological and subjective variables were collected at the end of the rest period and at 10-minute intervals throughout exercise.

### 2.8. Monocyte HSP72 Concentrations

An IgG1 isotype and concentration-matched FITC-conjugated negative control were used in order to assess nonspecific binding. Briefly, cells obtained after red cell lysis were fixed and permeabilised (AbD Serotec, UK) and a negative control (FITC, AbD Serotec, UK) or FITC-conjugated anti-HSP72 antibody (SPA-810, clone-C92F3A-5, Assay Designs, USA) was added to a final concentration of 100 *μ*g/mL; this was used to label 1 × 10^6^ cells according to the manufacturer's instructions and then incubated for 30 min in the dark. Samples were then analysed on a BD FACSCalibur (BD Biosciences) by flow cytometry with monocytes gated for forward/side scatter properties and further discriminated separately by CD14 expression in order to objectively determine the correct gate position for each participant sample. Mean florescence intensity (MFI) was calculated using CellQuest Pro software (BD Biosciences) with a total of 15000 cells counted. Fluorescence gained with the anti-HSP72 antibody divided by the fluorescence gained with the isotype-matched negative control. Results are reported as percentage change in MFI.

### 2.9. Circulating HSP72

eHSP72 was analysed with a preprepared enzyme-linked immunosorbent assay (ELISA) kit (StressExpress HSP72 ELISA kit, Stressgen Bioreagents, Canada). The HSP72 concentration was assessed via sample absorbance at 450 nm using a microplate reader (ELx800, BioTek Instruments, Inc., USA) and the KC Junior software package (v.1.41.3, BioTek Instruments, Inc., USA). A log-to-log scale of recombinant HSP72 standard concentration and absorbance measures were plotted to determine a line of best fit. The linear equation generated was then used to obtain inducible HSP72 concentration (ng·mL^−1^) from the absorbance of each sample. The sensitivity of the ELISA kit was 500 pg·mL^−1^ and both the inter- and intra-assay coefficient of variation was less than 10% (StressExpress HSP72 ELISA kit, Stressgen Bioreagents, Canada).

### 2.10. Data Analysis and Statistics

All statistical procedures were carried out using SPSS (version 20). Data are presented as means (SD) in the text and tables and as means (SD) in the figures. The primary outcome variables of interest in this experiment were the mHSP72 and eHSP72 responses to the HST.

One participant in the CON group was below the detection limit of the eHSP72 assay at rest throughout all trials and was removed from the analysis. Two members of STHA were below the detection limit of the eHSP72 assay prior to HA3 and were removed from the statistical analysis for the 3-day acclimation period.

All data was checked for skewness and kurtosis prior to analysis. A mixed model two-factor repeated measures ANOVA was used to make all group *x* time comparisons throughout each HST and to assess between and within group differences upon completion of the first and last acclimation day. *F* values were adjusted for sphericity where appropriate, and main and interaction effects were investigated by Tukey's HSD test. In order to investigate the relationship between preexercise and postexercise induced expression of mHSP72, a linear regression analysis was performed. Effect sizes for changes in mHSP72 were calculated using Cohen's *d* and used to compare the effectiveness of the STHA intervention with CON. The significance level for statistical tests was set at *P* < 0.05.

## 3. Results

### 3.1. Physiological and Perceptual Responses to the Acclimation Period

Participants in both groups were considered hydrated prior to each acclimation session with no differences in nude body mass (CON: day 1 = 75.8 ± 10.4 kg, day 2 = 75.8 ± 10.2 kg, day 3 = 75.9 ± 10.4 kg; STHA: day 1 = 77.4 ± 6.4 kg, day 2 = 77.4 ± 7.1 kg, day 3 = 77.3 ± 6.5 kg; *P* > 0.05) or USG (CON: day 1 = 1.009 ± 0.004, day 2 = 1.011 ± 0.007, day 3 = 1.008 ± 0.010; STHA: day 1 = 1.006 ± 0.005, day 2 = 1.010 ± 0.006, day 3 = 1.009 ± 0.008; *P* > 0.05) upon arrival to the laboratory each day. All participants in CON (*n* = 8) completed the full 60 minutes of cycling on each acclimation day. In STHA, 3 of 8 participants failed to complete the 60 minutes on day 1 (mean ± SD; 55.5 ± 6.2 mins) compared to 1 of 8 on the final day of acclimation (58.5 ± 4.2 mins). Mean and peak HR, *T*_rec_, *T*_skin_, *T*_body_, PSI, RPE, and TS were significantly higher during all 3 days of acclimation in STHA compared to the CON group (*P* < 0.01) (Table 1). Mean and peak HR, *T*_rec_, *T*_skin_, *T*_body_, PSI, RPE, and TS did not vary from HA1 to HA3 in either group (*P* > 0.05; Table 1). Sweat rates were unchanged throughout the acclimation period in CON (day 1 = 0.55 ± 0.18 L·hour^−1^, day 2 = 0.60 ± 0.33 L·hour^−1^, day 3 = 0.61 ± 0.38 L·hour^−1^; *P* > 0.05) and were higher in STHA compared to CON (*P* < 0.01). Sweat rate increased throughout the acclimation period in STHA (day 1 = 1.20 ± 0.46 L·hour^−1^, day 2 = 1.42 ± 0.74 L·hour^−1^, day 3 = 1.48 ± 0.5 L·hour^−1^; *P* < 0.05 versus HA1). Baseline plasma volumes were 52.9 ± 2.7% and 54.6 ± 2.9% in CON and STHA, respectively. Resting plasma volume was unchanged throughout acclimation in CON (day 2 = −1.1 ± 5.1%; day 3 = 1.0 ± 4.0%; *P* > 0.05). Plasma volume expansion was evident prior to HA3 in STHA; though this was highly variable (day 2 = 1.8 ± 3.9, day 3 = 4.6 ± 5.7%; *P* < 0.05).

### 3.2. mHSP72 Responses to the Acclimation Period

**Figure 2:**
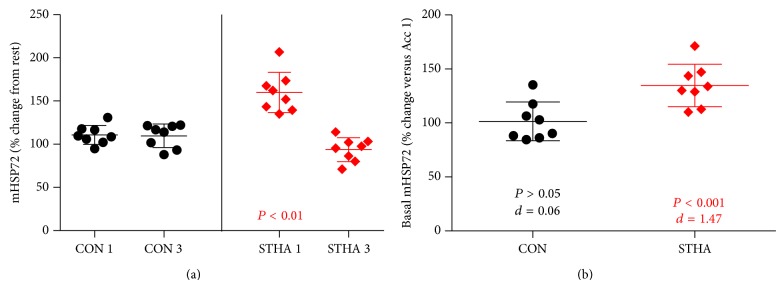
(a) Fold change in mHSP72 compared to resting baseline on intervention days 1 and 3 in the CON group (black circles) and STHA group (red diamonds). Post-HA1 mHSP72 was unaltered in the CON group and elevated in the STHA group (*P* < 0.01). Postexercise mHSP72 expression was attenuated following HA3 compared to postexercise HA1 in the STHA group (*P* < 0.05). (b) Basal mHSP72 remained unchanged throughout the acclimation period in the CON group (*P* > 0.05) and was elevated at baseline on HA3 in the STHA group (*P* < 0.001).

Resting mHSP72 did not vary between groups on HA1 (*P* > 0.05). mHSP72 increased immediately following HA1 STHA (58 ± 27%, *P* < 0.01, Figure 2(a)) but not CON (11 ± 11%, *P* > 0.05, Figure 2(a)). Resting mHSP72 was elevated from pre-HA1 to pre-HA3 in STHA (31 ± 23%, *P* < 0.001; *d* = 1.47, Figure 2(b)) and remained unchanged in CON (2 ± 18%, *P* > 0.05; *d* = 0.06, Figure 2(b)). mHSP72 was not elevated from rest following exercise on HA3 in either group (*P* > 0.05, Figure 2(a)). Postexercise mHSP72 on HA3 was lower compared to the postexercise data on HA1 for the STHA group (*P* < 0.05, Figure 2(a)). Regression analysis showed a negative relationship in the STHA group between preexercise expression and the magnitude increase (% change) in mHSP72 on HA1 (*R* ^2^ = −0.66, *P* = 0.014), which was weakened following HA3 (*R* ^2^ = 0.19, *P* = 0.278).

### 3.3. Circulating HSP72 Responses to the Acclimation Period

**Figure 3:**
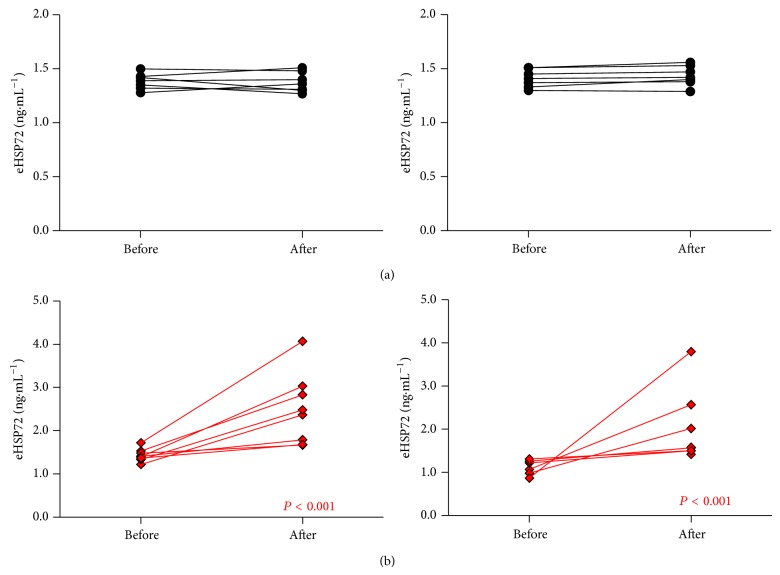
The intervention period did not induce any changes in postexercise eHSP72 in CON (black lines (a)). Postexercise eHSP72 was elevated from rest after exercise on HA1 and HA3 in the STHA group (*P* < 0.001, red lines (b)). Lines represent individual data.

eHSP72 remained unchanged from rest following exercise on each day of the 3-day protocol in the CON group (*P* > 0.05, Figure 3(a)). eHSP72 increased following exercise on day 1 and day 3 of the acclimation period (day 1: 1.06 ± 0.74 ng·mL^−1^; day 3: 1.04 ± 1.06 ng·mL^−1^; *P* < 0.001) in STHA (Figure 3(b)). Resting eHSP72 was lower (*n* = 6) on day 3 of STHA compared to day 1 (day 1: 1.43 ± 0.15 ng·mL^−1^; day 3: 1.12 ± 0.54 ng·mL^−1^), although this observation failed to reach statistical significance due to the high intersubject variability (*P* > 0.05; Figure 3(b)).

### 3.4. Physiological Responses to the Hypoxic Stress Test

**Figure 4:**
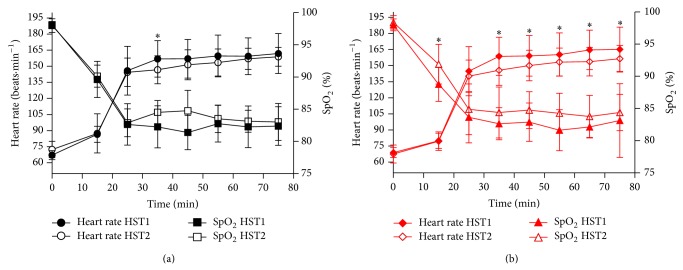
Heart rate was lower and SpO_2_ was higher between 20 and 30 minutes of exercise during HST2 in the CON group (a). However when an erroneous value from a participant is removed from the analysis no difference in SpO_2_ is present (see inset of (a)). Heart rate was lower and SpO_2_ higher in HST2 compared to HST1 for the STHA group throughout the exercise period (^*^ *P* < 0.05 versus HST1). Data are mean ± SD.

Heart rate was reduced in HST2 compared to HST1 (*P* = 0.019); however there was no trial *x* group interaction (*P* > 0.05). HR was lower in HST2 from HST1 at 20–30 minutes for CON (*P* < 0.05, Figure 4(a)) and 20–60 mins for STHA (*P* < 0.05, Figure 4(b)). SpO_2_ was higher between 20 and 30 mins in CON (*P* < 0.05, Figure 4(a)) and throughout exercise in HST2 compared with HST1 in STHA (*P* = 0.006, Figure 4(b)), although no trial *x* group interaction was observed (*P* > 0.05) (Figure 4). SpO_2_ data may have been affected by erroneous measurements during minutes 20 and 30 of HST2 in the CON group. One participant displayed fluctuating and unusually high SpO_2_ values in this time due to equipment malfunction, which was immediately corrected once identified and a replacement used. As this occurred during HST2 it was impractical to retest the participant. Removing the spurious data point affects the significance observed for SpO_2_ during the CON trial (*P* > 0.05).

### 3.5. Thermoregulatory Responses to the Hypoxic Stress Test

**Figure 5:**
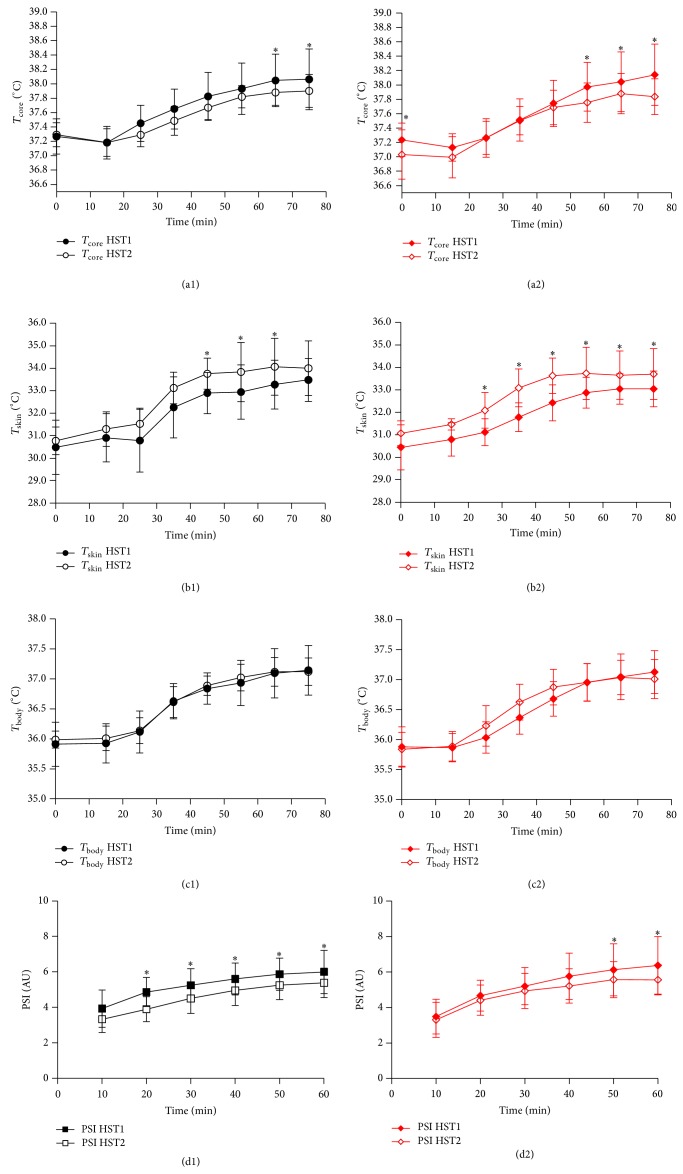
Thermoregulatory responses to the HST. *T*_core_ was lower in HST2 at 50–60 minutes in CON and from 40 minutes in STHA (*P* < 0.05). *T*_skin_ was elevated during HST2 from 30 to 50 minutes in CON and 20–60 minutes in STHA (*P* < 0.05). *T*_body_ was unchanged between HST1 and HST2. PSI was reduced from 20 to 60 minutes in CON and at 50 and 60 minutes in STHA (*P* < 0.05).

*T*_core_ was reduced and *T*_skin_ elevated during HST2 compared to HST1 for both experimental groups (*P* < 0.05; Figures 5(a1), 5(a2), 5(b1), and 5(b2)) whereas *T*_body_ did not vary between HST1 and HST2 (*P* > 0.05; Figures 5(c1) and 5(c2)). No trial *x* group interaction was found for rectal, mean skin, or mean body temperature (*P* > 0.05). Physiological strain was reduced during HST2 compared to HST1 in both groups (*P* < 0.01; Figures 5(d1) and 5(d2)) with no trial *x* group interaction observed (*P* > 0.05; Table 2).

### 3.6. Subjective Responses to the HST

RPE (*P* = 0.04) and TS (*P* = 0.02) were lower during HST2 compared to HST1 for both groups in comparison to HST1, with no trial *x* group interaction being observed (*P* = 0.17).

### 3.7. mHSP72 Responses to Hypoxic Stress Test

**Figure 6:**
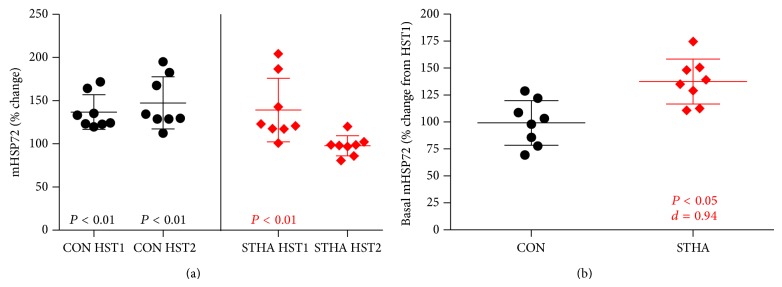
(a) Fold change in mHSP72 compared to resting baseline on HST1 and HST2 in the CON group (black circles) and STHA group (red diamonds). Post-HST1 mHSP72 elevated from baseline in both experimental groups (*P* < 0.01). mHSP72 was elevated from rest after HST2 in the CON group (*P* < 0.01) and attenuated in the STHA group. (b) Basal mHSP72 remained unchanged between HST1 and HST2 in the CON group (*P* > 0.05) and was elevated prior to HST2 in the STHA group (*P* < 0.05, *d* = 0.94).

**Figure 7:**
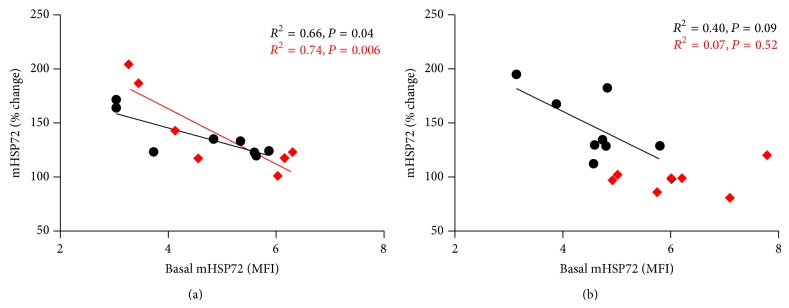
Regression analysis between preexercise monocyte expression of mHSP72 and the fold change (%) in mHSP72 following HST1 (a) and HST2 (b). Black circles denote CON and red diamonds denote STHA. Prior to HST1 the percentage change in mHSP72 after exercise had an inverse relationship with basal mHSP72. This feature was present in both CON (black circles) and STHA (red diamonds). After the intervention period the inverse relationship was still present in the CON (b), but no longer a feature of the STHA group, possibly as a result of the increase in basal mHSP72 observed prior to onset of HST2 (Figure 6(b)).

The initial HST produced an increase in mHSP72 in CON (34 ± 51%) and STHA (39 ± 37%). This response was not different between groups (*P* > 0.05). Resting mHSP72 was elevated from HST1 to HST2 in STHA (28 ± 26%, *P* < 0.05; *d* = 0.94) and unchanged for CON (3 ± 27%; *d* = −0.08). The mHSP72 response to HST2 was similar to HST1 in the CON group (48 ± 30%). STHA attenuated in post-HST2 mHSP72 expression compared to HST1 (98 ± 12%; *P* < 0.05) and was lower after exercise in STHA compared to CON (*P* < 0.05; *d* = −0.45) (Figure 6). Significant correlations were observed for the preexercise mHSP72 expression and the percentage change in expression following exercise for HST1 in both the CON and STHA groups (Figure 7(a)) and were also present during HST2 for the CON group (Figure 7(b)). This relationship was weakened for HST2 in the STHA group (Figure 7(b)).

### 3.8. Circulating HSP72 Responses to the Hypoxic Stress Test

**Figure 8:**
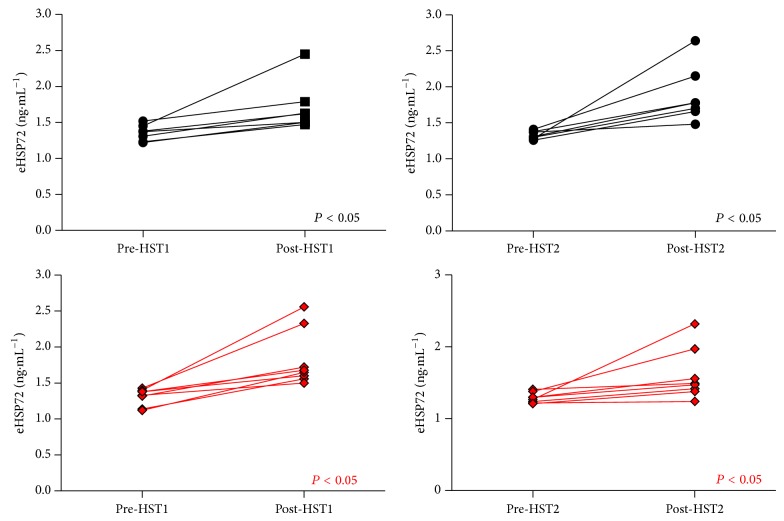
eHSP72 before and immediately after HST1 and HST2. Lines represent individual participants. Postexercise eHSP72 was elevated after exercise in both groups following each HST.

eHSP72 increased to a similar magnitude following HST1 and HST2 in the CON group (HST1: 0.35 ± 0.29 ng·mL^−1^; HST2: 0.55 ± 0.40 ng·mL^−1^) and STHA group (HST1: 0.51 ± 0.35 ng·mL^−1^; HST2 0.32 ± 0.34 ng·mL^−1^; Figure 8). The eHSP72 response to the HST1 was smaller and less variable compared to the response to an acute heat stressor (HA1: 1.06 ± 0.74 ng·mL^−1^; HST1 0.51 ± 0.35 ng·mL^−1^).

## 4. Discussion

To the author's knowledge this is the first* in vivo* human study examining the phenomenon of cross acclimation between heat and hypoxic stressors during the initial phase of heat acclimation. The key findings of the study were that 3 daily exercise-heat exposures were sufficient to increase basal mHSP72 stores. The increase in basal mHSP72 observed prior to HA3 was present prior to the onset of HST2 in the STHA group resulting in an attenuation of hypoxia mediated mHSP72 expression after exercise. These results support the experimental hypothesis and indicate that STHA has potential for improving cellular tolerance to acute hypoxic exercise.

### 4.1. Attainment of Heat Acclimation

An important methodological aspect of the present study was the capacity of 3 days of repeated heat exposures in initiating heat acclimation adaptations. The STHA group displayed the classically described reductions in exercising HR and exercising core temperature, reduced overall physiological strain, and increased sweat rates (Table 1). The magnitudes of peak exercising reductions in the STHA group for HR (~7 beats·min^−1^), *T*_core_ (~0.3°C), and PSI (~0.5 A.U) are less than those observed following an identical acclimation protocol in similarly trained participants (~14 beats·min^−1^; 0.4°C; 1.6 A.U; [31]). Our results are similar to those reported by Marshall et al. ([14]; ~9 beats·min^−1^, 0.2°C, 0.7 A.U) in participants that were described as heat acclimated following 3 repeated heat exercise exposures. This suggests that our STHA group was in the initial phases of heat acclimation.

### 4.2. Monocyte and Circulating HSP72 Responses to Heat Acclimation

Prior to the commencement of STHA (7 days after HST1), basal mHSP72 values had returned to those observed before HST1 in both groups. This is experimentally important, as the magnitude of HSP72 response to a stressful insult has been shown to be proportional to its basal content prior to stressful insults [32]. mHSP72 was increased from baseline following the initial day of heat exposure in the STHA group as previously observed following acute heat exposure (Figure 2(a); [33]). The mHSP72 response occurred to a similar magnitude when sampled at similar time points to those reported in other studies [33, 34]. After 2 acclimation days resting mHSP72 remained elevated (30 ± 23%; *d* = 1.47, Figure 2(b)) compared to values recorded at rest on day 1 of the acclimation period. This is similar to data reported in previous studies. For example, a 40% increase in basal lymphocyte HSP72 has been observed after 4 days of walking for 90 minutes in 33°C, 30–50% humidity [35], and increases of ~30% in mHSP72 MFI were observed 24 hours after 60 minutes of running in hot conditions (60 minutes at 90% of lactate threshold velocity, 28°C; [33]). A negative relationship between basal mHSP72 and the magnitude of postexercise change was observed in the current study on the first day of the HA period (*r* = −0.81, *P* = 0.014). This is in line with the accepted inverse relationship between basal mHSP72 and its induction via a stressor [32]. Following 3 days of exercising heat exposure this relationship was weakened (*r* = −0.44, *P* = 0.28). The increase in basal levels during the present investigation blunted the mHSP72 response following HA3 (94 ± 14%, Figure 2(a)), which is an accepted characteristic in the shift towards a heat acclimated state [1, 15, 36] and indicative of increased cellular tolerance [6, 36].

Resting eHSP72 has been shown to decrease following the initial 2 days [2], 5 days [37], and 11 days [36] of exercise-heat acclimation. Our data appear to follow this trend (Figure 3(b)) but did not reach statistical significance. Two participants in the heat group recorded levels below the detection level of the assay at rest on HA3. It is worth noting that these participants displayed the largest postexercise changes in eHSP72 immediately after HA3. This follows the observed trend in intracellular mHSP72 responses in that greater magnitudes in expression are observed in those with lower basal values. It is unclear whether a similar relationship exists for eHSP72 release. Postexercise eHSP72 was still significantly increased from rest on day 3, though the magnitude of this increase was smaller. The inhibition of eHSP72 release/production was also observed following a 5-day HA protocol, but only in participants that displayed classical signs of heat acclimation (reduced exercise HR and *T*_core_; [37]). A similar response was observed after a heat stress test (90 minutes running at 50% $\dot{\text{V}}{\text{O}}_{2\text{peak}}$ using a controlled hyperthermia protocol) after 11 days of heat acclimation [36]. The release of eHSP72 has been shown to be both intensity and duration dependent [13] and also requires a minimum level of external stress to induce its appearance/release [38]. The 3-day STHA period in this present study reduced the level of both thermal and cardiovascular strain, as evidenced by the reduced exercising HR and *T*_rec_, (Table 1); thus it is possible that the external conditions experienced by participants on HA3 were no longer sufficient to activate a similar response in eHSP72. That the CON group displayed minimal changes to these variables indicates that it is reasonable to conclude that the heat load and the consequent increase in physiological strain experienced by the STHA group were of sufficient magnitude to induce a shift towards the acclimated phenotype.

### 4.3. Monocyte HSP72 Responses to the Hypoxic Stress Test

Cross-tolerance between heat acclimation and oxygen deprivation stressors is well documented within animal models [39, 40]. In accordance with previous research [3, 8, 41], mHSP72 increased following the first acute hypoxia exposure in both CON (33 ± 37%; Figure 6(a)) and STHA (39 ± 17%; Figure 6(a)). A similar magnitude in mHSP72 response was observed following an identical hypoxic challenge within our laboratory [29]. It is likely that the increased oxidative stress associated with acute hypoxia and the subsequent damage to membrane structures and proteins, while activating apoptotic pathways, act as stimuli for HSP72 induction during hypoxia [3, 42, 43]. That the postexercise values reported in this present study are lower than those reported by an acute-resting intervention [3, 8] is likely to be due to the different participant characteristics and large interindividual variation in the mHSP72 response to stressors.

The STHA induced increase in mHSP72 persisted for at least 48 hours after the final HA session in the STHA group. Basal mHSP72 was elevated prior to HST2 (28 ± 26%) when compared to the preexercise levels before HST1 (Figure 6(b)). This prolonged elevation in mHSP72 after removal from repeated daily stress exposures has been observed 48 hours after 10 consecutive daily, 75-minute passive exposures to hypoxia in healthy humans [41]. The authors observed increases in mHSP72 of ≈30% per day for the first 5 days of daily repeated hypoxic exposure, decreasing to 16% per day for the final 5 days, representing a plateau in the response of mHSP72. Thus a total ≈200% increase in mHSP72 over the 10-day period, which remained elevated 48 hours after the final exposure (≈225% [41]). However during the initial 3 days of the hypoxic acclimation period, mHSP72 was increased by ≈50% from baseline. The magnitude of mHSP72 induction following the early stages of repeated hypoxic exposures is not dissimilar to that seen in the current experiment as a response to repeated exercising heat exposure (≈30% increase in baseline on day 3). It is worth noting that participants in the current investigation began to show a blunting in the mHSP72 on the third day of acclimation, whereas Taylor et al. [41] demonstrated continual, modest increases in mHSP72 following passive hypoxic exposures on days 4, 5, and 10. This indicates that the internal strain placed on participants in the present investigation may have reached an earlier ceiling for the level of strain required to produce further mHSP72. Increased basal HSP72 is a well-defined characteristic of both acquired thermotolerance [35, 44] and improved cellular tolerance to repeated hypoxic exposures [8, 41] in humans. Thus it is not surprising that a stressor that invokes the HSR and leads to the subsequent increase in basal mHSP72 would lead to improved cellular tolerance to a second, novel stressor, in this instance acute hypoxia.

The mHSP72 response to the HST was attenuated after STHA with a large and significant effect observed in the STHA group (*d* = −0.45, *P* < 0.05; Figure 6(a)). The mechanism by which an increase in basal levels of mHSP72 may inhibit its own expression is related to HSP72 binding to heat shock transcription factor 1 (HSF1). In unstressed cells HSF1 is bound to HSP72. Under stressful conditions HSP72 binds to denatured proteins, freeing HSF1. HSF1 trimerises and relocates to the nucleus where it binds to the heat shock element (HSE), initiating transcription of HSP72. When sufficient HSP72 has been produced to deal with the rigors of the stressor, HSP72 rebinds to the HSF and halts further transcription [4]. It is possible that STHA induced increases in mHSP72 elevate the cellular stress required to induce further HSF1 activation. The induction of mHSP72 via a STHA period was sufficient to allow the cells to cope with the hypoxic challenge, maintaining normal cell function and homeostasis. The authors do not suggest whole body preconditioning and cellular tolerance has been conferred from the initial phase of acclimation studied and subsequent elevations in one marker of cellular stress. Without parallel measures in skeletal muscle, the whole body responses during the initial phase of acclimation cannot be fully explored, and thus this response requires further investigation. The inclusion of a normothermic-exercise control group allowed for the effects of exercise and heat to be separated, which has been a design issue with other studies investigating the heat shock response in humans. Exercise in the absence of an external heat stress led to small nonsignificant (10%; Figure 2(a)) increases in mHSP72, similar to those previously reported for similar work bouts [45]. It is possible that the increased physiological strain observed in the STHA group, and not the imposition of heat* per se*, drove the cross acclimatory affect [46]. No attempt was made to ensure that each group was achieving similar exercising heart rates; thus the increased exercise intensity in the STHA group may in part have elicited the HSR and alterations in physiological function. The work rate utilized (50% $\dot{\text{V}}{\text{O}}_{2\text{peak}}$) in the present investigation has been shown to allow participants to remain below the individual anaerobic threshold when exercising in both normothermic and hot (40°C) conditions [47]. In addition, individual $\dot{\text{V}}{\text{O}}_{2\text{peak}}$ in 40°C conditions has been shown to decline by ~5% compared to $\dot{\text{V}}{\text{O}}_{2\text{peak}}$ achieved in normothermic conditions in similarly trained individuals compared to those used in the present investigation [47]. Therefore it is likely that the metabolic stress presented by the work intensity and differences in the relative workloads was not significantly different between the conditions, with heat being the mediating factor for observed experimental effects. The inclusion of a hypoxic exercise group in future studies would also allow differences in expression kinetics to be quantified by the two divergent physiological stressors and a further exploration of heat-mediated tolerance to hypoxia.

### 4.4. Circulating HSP72 Responses to the HST

Plasma HSP72 also increased following the HST in both sets of participants (Figure 8). This is the first study to measure eHSP72 in response to an acute exercising hypoxic exposure in humans. eHSP72 increased significantly after HST1 in each experimental group (Figure 8), whereas normoxic exercise failed to induce any change in this variable in the control group during the intervention period (Figure 3(a)). It is likely that the normoxic exercise challenge failed to invoke a significant endogenous stress in order to stimulate the release of eHSP72 in this group. In contrast to this, the acute exercising heat challenge experienced on HA1 by the heat group produced a significantly greater increase in this biomarker than which was seen in response to the initial HST. This would suggest that the level of thermal strain experienced by this group presented a greater physiological stress than experienced during the level of acute hypoxia studied in this investigation. This is perhaps not surprising, as the rate of core temperature increase and delta change in core temperature have been found to be important external moderators in altering eHSP72 expression [38]. However, while these are important factors invoking a change in circulating levels of this protein, other factors have also been shown to be important. For example, both intensity and duration of exercise affect eHSP72 concentrations when work is performed in thermoneutral conditions [13, 33], with the addition of a thermal stressor increasing this response [2, 14, 48]. Therefore the increase in eHSP72 following HST1 may reflect the increase in relative work intensity. The absolute level of work used (50% normoxic $\dot{\text{V}}{\text{O}}_{2\text{peak}}$) has been shown in our laboratory, using participants of similar physiological characteristics and training background, to correspond to 78% of hypoxic $\dot{\text{V}}{\text{O}}_{2\text{peak}}$ [47]. In conditions of matched heat stress (40°C, 50%RH) but differing workloads (60 and 75% $\dot{\text{V}}{\text{O}}_{2\text{peak}}$), no difference in postexercise eHSP72 was observed, despite markedly different times to exhaustion (60%: 58.9 ± 10.9 minutes; 75%: 27.2 ± 9.0 minutes) [13]. The magnitude of eHSP72 increase following the HST was lower than observed by Fulco et al. [49] who reported an increased eHSP72 of approximately 2 ng·mL^−1^. In order to focus on the specific effects of hypoxia* per se* on eHSP72, matching both absolute and relative levels of work would be required in both normoxic and hypoxic conditions. This was beyond the scope of this present investigation; thus the response of eHSP72 to acute bouts of moderate hypoxia therefore warrants further investigation.

### 4.5. Physiological Responses to the HST

It is relatively common for both athletes and military personal to be exposed to moderate altitude and be expected to perform physical tasks without undergoing prior acclimatization. It is well established that, even in the moderate altitude conditions studied herein, exercise performance, psychomotor performance, and cognitive function are reduced [49–51]. Adaptation to altitude requires approximately 14 days of residence, with molecular adaptations serving to improve oxygen delivery to cells as well as maintain the structure and function of cells and organs [6]. However, in scenarios where the rapid deployment of troops is necessary the extended altitude acclimation time frame poses logistical problems. From a practical perspective, interventions which can maintain or improve performance at altitude are therefore of interest. Heat acclimation reduces oxygen uptake, induces glycogen sparing, increases plasma volume, and improves myocardial efficiency and contraction, thereby reducing the stress on the cardiovascular system for a fixed level of work [52–54]. Despite the hematological and respiratory mechanisms of adaptation differing between heat and hypoxia, the increased physiological efficacy that is seen following a period of heat acclimation [55] coupled with the shared heat and hypoxic molecular adaptations of the HSP network may point to a cross acclimation effect being attainable. However experiments that have explored the cellular and molecular responses to preconditioning and cross acclimation interventions have done so without due consideration of the whole body physiological implications arising from any observed adaptation or increased cellular tolerance [8]. This study attempted to determine if any heat acclimation-induced alterations in the cellular stress response elicited measureable improvements in physiological tolerance when exposed to a subsequent, acute exercising hypoxic challenge. The findings of this present investigation point to the possibility that a prior period of exercise-heat stress may be associated with beneficial physiological outcomes when later exposed to a period of acute hypoxic work. The reduced exercising HR in the STHA group, combined with an elevated SpO_2_ (Figure 4), would indicate that this group was more tolerant to the acute hypoxia after the acclimation period. The reduction in SpO_2_ may have been related to the reduced body temperature in the STHA producing a leftward shift in the O_2_ dissociation curve. These preliminary results indicate that further work examining heat acclimation and hypoxic performance is warranted. The reductions in mean and peak exercising HR of ~9 beats·min^−1^ may indicate an increased capacity for work in these conditions; however follow-up studies involving a hypoxic-adaptation group would allow the efficacy of time matched acclimation protocols to be assessed. Furthermore, a physical performance test before and after intervention would determine whether the reductions in HR and improved cellular tolerance observed herein following STHA can enhance physical performance in hypoxic conditions. It would also be of interest to determine the “decay rate” of the acclimation and cross acclimation affect during both short-term (<5 days) and long-term acclimation protocols in order to optimize the time frame for exposure to secondary stressors, a methodological consideration that was not practicable in the present investigation.

## 5. Conclusion

In conclusion, STHA consisting of 3 consecutive exercise-heat exposures resulted in increased levels of monocyte HSP72 in humans and affected the expression characteristics of this protein during the acclimation period and in subsequent exposure to acute normobaric hypoxic exercise. The improved capacity of the chaperone system may have attenuated the cellular stress response to subsequent hypoxia and warrants additional investigation. Additionally, small yet significant changes in cardiovascular and thermoregulatory responses to subsequent hypoxia were evident in the STHA compared to CON. These responses also warrant further investigation during different phases of the acclimation process.

## Figures and Tables

**Table 1:** Peak and mean exercising (mean ± SD) physiological and thermoregulatory measures during the 3-day acclimation period tests for the control (CON) and short-term heat acclimation (STHA) groups.

Measure	Change in NBM (kg)	Peak HR (bts·min^−1^)	Mean HR (bts·min^−1^)	Peak *T*_core_ (°C)	Mean *T*_core_ (°C)	Peak *T*_skin_ (°C)	Mean *T*_skin_ (°C)	Peak *T*_body_ (°C)	Mean *T*_body_ (°C)	Peak PSI (A.U)	Mean PSI (A.U)
CON											
Day 1	0.6 ± 0.2	151 ± 21	144 ± 18	38.0 ± 0.2	37.7 ± 0.5	33.8 ± 1.3	33.0 ± 1.0	37.2 ± 0.2	36.8 ± 0.2	5.5 ± 1.1	4.6 ± 0.8
Day 2	0.6 ± 0.3	151 ± 23	142 ± 19	38.0 ± 0.2	37.7 ± 0.5	33.7 ± 1.0	32.6 ± 1.3	37.0 ± 0.5	36.7 ± 0.2	5.6 ± 1.3	4.5 ± 0.8
Day 3	0.6 ± 0.4	149 ± 23	143 ± 20	38.1 ± 0.4	37.7 ± 0.2	33.6 ± 0.9	32.8 ± 0.7	37.2 ± 0.3	36.7 ± 0.3	5.7 ± 1.4	4.5 ± 0.9
STHA											
Day 1	1.2 ± 0.5^*^	180 ± 13^*^	165 ± 14^*^	38.8 ± 0.3^*^	38.1 ± 0.2^*^	36.6 ± 1.0^*^	35.9 ± 1.0^*^	38.3 ± 0.3^*^	37.6 ± 0.3^*^	8.3 ± 1.0^*^	6.0 ± 0.8^*^
Day 2	1.4 ± 0.5^*^	176 ± 13^*^	162 ± 14^*^	38.7 ± 0.4^*^	38.0 ± 0.2^*^	36.6 ± 0.9^*^	36.1 ± 0.4^*^	38.3 ± 0.4^*^	37.6 ± 0.2^*^	8.1 ± 1.2^*^	5.9 ± 0.9^*^
Day 3	1.5 ± 0.5^*^	173 ± 13^*^	160 ± 13^*^	38.5 ± 0.3^*^	37.8 ± 0.1^*^	36.2 ± 1.0^*^	35.7 ± 0.7^*^	38.1 ± 0.4^*^	37.4 ± 0.3^*^	7.8 ± 1.1^*^	5.7 ± 0.8^*^

NBM = nude body mass; PSI = physiological strain index.^*^Difference between experimental groups (*P* < 0.01).

**Table 2:** Peak and mean exercising (mean ± SD) physiological and thermoregulatory measures for the pre- (HST1) and postacclimation (HST2) hypoxic stress tests for the control (CON) and short-term heat acclimation (STHA) groups.

Measure	Change in NBM (kg)	Peak HR (bts·min^−1^)	Mean HR (bts·min^−1^)	Peak *T*_core_ (°C)	Mean *T*_core_ (°C)	Peak *T*_skin_ (°C)	Mean *T*_skin_ (°C)	Peak *T*_body_ (°C)	Mean *T*_body_ (°C)	Peak PSI (A.U)	Mean PSI (A.U)
CON											
HST1	0.45 ± 0.3	162 ± 19	157 ± 15	38.1 ± 0.4	37.8 ± 0.4	33.5 ± 1.2	32.6 ± 1.0	37.2 ± 0.4	36.8 ± 0.5	6.0 ± 1.2	5.3 ± 1.2
HST2	0.50 ± 0.2	160 ± 8^*^	154 ± 12^*^	37.9 ± 0.3^*^	37.8 ± 0.3^*^	34.0 ± 1.0^*^	33.4 ± 1.3^*^	37.3 ± 0.1	36.9 ± 0.4	5.8 ± 0.8^*^	4.9 ± 1.1^*^
STHA											
HST1	0.51 ± 0.2	165 ± 20	159 ± 20	38.1 ± 0.4	37.8 ± 0.4	33.1 ± 0.8	32.4 ± 0.5	37.1 ± 0.4	36.7 ± 0.5	6.4 ± 1.6	5.3 ± 1.5
HST2	0.81 ± 0.2	156 ± 12^*^	150 ± 14^*^	37.8 ± 0.3^*^	37.6 ± 0.3^*^	33.7 ± 1.3^*^	33.3 ± 1.1^*^	37.0 ± 0.3	36.8 ± 0.4	5.6 ± 0.9^*^	4.8 ± 1.2^*^

^*^Difference between HST1 and HST2 (*P* < 0.05).
